# Ultrasonically assisted fabrication of electrochemical platform for tinidazole detection

**DOI:** 10.1016/j.ultsonch.2024.107056

**Published:** 2024-09-01

**Authors:** Chaojun Zhang, Rui Liu, Rijia Liu, Wenyu Cui, Yuan Sun, Wein-Duo Yang

**Affiliations:** a Center of Pharmaceutical Engineering and Technology, Harbin University of Commerce, Harbin 150076, China; b School of Pharmacy, Harbin University of Commerce, Harbin 150076, China; c Department of Chemical and Materials Engineering, National Kaohsiung University of Science and Technology, Kaohsiung 80778, Taiwan

**Keywords:** Bimetallic oxide, g-C_3_N_4_, Electrochemical detection, Electrochemical sensor, Tinidazole

## Abstract

Based on sonochemistry, green synthesis methods play an important role in the development of nanomaterials. In this work, a novel chitosan modified MnMoO_4_/g-C_3_N_4_ (MnMoO_4_/g-C_3_N_4_/CHIT) was developed using ultrasonic cell disruptor (500 W, 30 kHz) for ultra-sensitive electrochemical detection of tinidazole (TNZ) in the environment. The morphology and surface properties of the synthesized MnMoO_4_/g-C_3_N_4_/CHIT electrode were characterized using X-ray diffraction (XRD), fourier transform infrared spectroscopy (FT-IR), scanning electron microscope (SEM) and transmission electron microscope (TEM). Cyclic voltammetry (CV) and differential pulse voltammetry (DPV) techniques were utilized to assess the electrochemical performance of TNZ. The results indicate that the electrochemical detection performance of TNZ is highly efficient, with a detection limit (LOD) of 3.78 nM, sensitivity of 1.320 µA·µM^−1^·cm^−2^, and a detection range of 0.1–200 μM. Additionally, the prepared electrode exhibits excellent selectivity, desirable anti-interference capability, and decent stability. MnMoO_4_/g-C_3_N_4_/CHIT can be successfully employed to detect TNZ in both the Songhua River and tap water, achieving good recovery rates within the range of 93.0 % to 106.6 %. Consequently, MnMoO_4_/g-C_3_N_4_/CHIT’s simple synthesis might provide a new electrode for the sensitive, repeatable, and selective measurement of TNZ in real-time applications. Using the MnMoO_4_/g-C_3_N_4_/CHIT electrode can effectively monitor and detect the concentration of TNZ in environmental water, guiding the sewage treatment process and reducing the pollution level of antibiotics in the water environment.

## Graphical abstract

**Figure:**
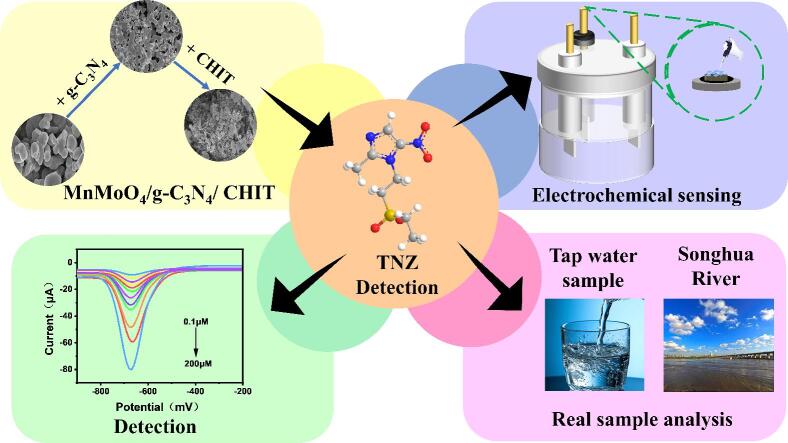

## 1 Introduction

The fabrication of novel nanomaterials using sonochemical methods is an attractive option due to their non-toxicity and environmental friendliness. Sonochemical reactions rely on the effects of cavitation and activation, which involve the formation, expansion, and collapse of bubbles that generate pulses of high temperature and pressure. These conditions, combined with vigorous micro-mixing, can result in the creation of nanomaterials with unique properties. Sonochemical methods offer several advantages as compared to other synthesis approaches. They allow the convenient synthesis of the doped nanomaterials without the use of toxic reagents or chemicals, eliminate the need for the additional reducing agents, and enable the rapid reduction rates. By incorporating suitable stabilizers, this method can produce extremely small nanoclusters. The ultrasonic method can also induce vibration and agitation among the molecules in the solution, thereby promoting the formation of crystals. This vibration and agitation can disrupt intermolecular forces in the solution, aiding in the formation of new crystal nuclei and promoting crystal growth. Besides, ultrasonic waves can alter the growth direction and the rate of crystals, thus influencing their morphology, size, and crystal structure. Through this mechanism, ultrasonic waves can enhance the purity and crystallinity of the crystals, resulting in a more perfect and uniform final product. Overall, sonochemical methods can provide new insights for the synthesis of nanomaterials [1].

Metal molybdates QMoO_4_ (Q=Fe, Co, Mn, Ni, Zn, etc.) have garnered substantial attention from researchers owing to their sturdy crystal structures, redox characteristics, remarkable physicochemical properties, and superior charge carrier mobility [2], [3]. Due to the ability of metal ions to exist in the multiple oxidation states, diverse methods have been developed for the synthesis of metal molybdate compounds. These compounds have been extensively studied as the potential electrode materials in various fields. Molybdates, when combined with the first-row transition metals, form a fascinating class of compounds due to their enhanced alkalinity and chemical stability. Of the particular interest are composite materials incorporating molybdenum and manganese, which display the exceptional improvements in both conductivity and redox activity [4]. MnMoO_4_ exhibits superior structural stability compared with the cobalt and nickel-based molybdates, due to its distinctive structural properties and lower cohesive energy. The trinary manganese molybdate (MnMoO_4_) emerges as a promising material for electrochemical applications because of its myriad benefits, including a broad inherent working voltage range, excellent retention capacity, cost-effectiveness, low toxicity, abundance in nature, stable crystal structure, high conductivity, high theoretical specific capacitance, environmental sustainability, exceptional catalytic performance, efficient operation across a wide pH range, and outstanding cycling stability [5], [6], [7]. In recent years, researchers have undertaken numerous efforts to integrate MnMoO_4_ with a variety of materials. For instance, the combination of MnMoO_4_ and MXene has been explored for the detection of hydroquinone (HQ) and catechol (CC) [8]. The MnMoO_4_-MXene-modified glassy carbon electrode (GCE) sensor exhibits a wide linear response range for both hydroquinone and catechol, spanning from 5 nM to 65 nM. Moreover, the sensor demonstrates detection limits of 0.26 nM for hydroquinone and 0.30 nM for catechol. Furthermore, MnMoO_4_ has been synthesized as a MoS_2_/MnMoO_4_@Ti nanocomposite for hydrogen production [9]. MnMoO_4_ was integrated with g-C_3_N_4_ and CNT to create MnMoO_4_@g-C_3_N_4_/CNT for the development of a hybrid capacitive deionization (HCDI) system. Exhibiting a maximum specific adsorption capacity (SAC) of 42.6 mg/g and a retention rate of 91 % over the ten consecutive charge–discharge cycles, the system showcased the exceptional cycle stability of the MnMoO_4_@g-C_3_N_4_/CNT electrode [10]. Owing to its outstanding characteristics encompassing remarkable mechanical and electrical performance, g-C_3_N_4_ has recieved growing attention as a two-dimensional semiconductor material subsequent to the discovery of graphene [11]. g-C_3_N_4_ has been demonstrated to enhance electron transfer reactions of the target molecules. Its capacity to absorb visible light, thermal stability, exceptional chemical stability, non-toxic nature, abundant light absorption capabilities, and the straightforward fabrication process bestow upon it vast potential across diverse application domains [12], [13]. Nevertheless, the inherent conductivity of g-C_3_N_4_ is generally low, restricting its effectiveness and efficacy in the electrochemical applications. Chitosan (CHIT), characterized by its active hydroxyl and amino groups, demonstrates notable film-forming properties, superior adhesion, and biocompatibility. Consequently, CHIT has found widespread application across several environmental sectors. Moreover, a variety of materials, including nanoparticles, composite materials, metal composites, carbon nanotubes, and graphene oxide, are integrated into CHIT polymers to enhance their electrical conductivity [14].

Tinidazole (TNZ) (1-(2-ethylsulfonyl ethyl)-2-methyl-5-nitroimidazole) is a commonly used antibiotic in Europe and developing countries for the treatment of various amoebic and parasitic infections [15]. TNZ can also be utilized to address specific bacterial infections, such as intestinal or vaginal infections [16]. TNZ can be employed in the treatment of trichomoniasis, a sexually transmitted disease affecting both men and women. The mechanism of action of nitroimidazole drugs involves the disruption of DNA by means of nitro group metabolism. The production of free radicals due to this reduction process may contribute to the cytotoxic effects. The excessive presence of tinidazole in the environment can pose several hazards. Firstly, its discharge into water bodies may elicit toxic effects on the aquatic organisms, disrupting the aquatic ecosystem balance. Secondly, the excessive tinidazole entering the soil can disturb the soil microbial ecosystem and impede plant growth. Furthermore, as an antibiotic, the excessive use of tinidazole can lead to microbial resistance in the environment, thereby reducing its efficacy in the medical applications. The long-term exposure to high concentrations of tinidazole may pose risks to human health. According to the information provided by the WHO, the acute oral LD_50_ (lethal dose for 50 % of the population) of tinidazole in rats is approximately 3.5–4.0 g/kg, and in mice, it is about 2.0–2.5 g/kg. For humans, the recommended therapeutic dose of tinidazole is generally a single oral dose of 2.0 g, which can be used to treat certain acute infections. The prolonged or the excessive use may lead to the severe adverse effects, including neurological symptoms and potential carcinogenic effects [17].

Various analytical methods have been documented for detecting TNZ, including high-performance liquid chromatography (HPLC) [18], liquid chromatography-mass spectrometry (LC-MS) [19], gas chromatography-mass spectrometry (GC–MS) [20], capillary electrophoresis (CE), and chemiluminescence. While these methods ensure accuracy, they are plagued by the operational complexities, the lengthy analysis times, the costly instrumentation, and the need for skilled operators. In contrast, the electroanalytical techniques stand out for their affordability[21], [22], ease of use, high sensitivity, exceptional selectivity, portability, minimal sample consumption, and simplicity in downsizing.

Electrochemical sensors are extensively utilized across different sectors, such as environmental analysis, food safety, clinical diagnostics, and public health [23]. Apart from antibiotics, these sensors are capable of detecting a diverse range of the target analytes, including proteins, metabolites, neurotransmitters, electrolytes, and heavy metals. Functioning by converting electric signals generated during the analyte reactions into the measurable signals directly correlated to the analyte concentration, the electrochemical sensors are highly sensitive. This sensitivity makes them valuable for the antibiotic detection, handling small sample masses and volumes accurately, and ensuring selectivity [24]. Moreover, these electrochemical sensors offer numerous advantages, such as straightforward measurement procedures, rapid response times, low energy consumption, linear output, repeatability, accuracy, affordability, and exceptional sensitivity and selectivity [25]. These characteristics establish them as indispensable tools for various academic research pursuits. Consequently, this study asserts that the electrochemical methods stand out as the preferred approach for the TNZ determination in samples.

The powerful cavitation effects generated by ultrasonic waves in liquids can cause intense oscillation and hydrodynamic effects in solvents. This effect helps to effectively disperse solid particles, nanoparticles, or other solutes in solvents, forming a suspension system. This increases the contact area between reactants, promoting reaction rates and enhancing reaction uniformity and efficiency. Moreover, compared with the traditional two-dimensional nanosheet synthesis and preparation methods, ultrasonic waves accelerated the insertion of MnMoO_4_ into g-C_3_N_4_. These binary g-C_3_N_4_ nanosheets also exhibited higher efficiency in the electrocatalytic reactions, extending their utility in electrochemical sensor applications. Thus, in this study, the MnMoO_4_/g-C_3_N_4_/CHIT composite was synthesized using the ultrasonic methods for detecting TNZ. As far as we konw, this is the first time MnMoO_4_/g-C_3_N_4_/CHIT has been employed for the detection of TNZ. Differential pulse voltammetry (DPV) was used to evaluate the modified electrode and estimate its limit of detection (LOD) for the TNZ detection. Due to its non-hydrophobic, hydrogen bonding, and stable interactions, the prepared electrode can be used for the detection of TNZ in complex environments such as river water and tap water. The sensor exhibits high sensitivity, rapid response time, excellent repeatability, low detection limit, and resilience to interference from potential contaminants.

## 2 Materials and methods

### 2.1 Reagents

Urea (CH_4_N_2_O,), Potassium Ferricyanide (K_3_[Fe(CN)_6_]), Potassium Ferrocyanide (K_4_[Fe(CN)_6_]), Manganese Chloride Tetrahydrate (MnCl_2_·4H_2_O), Sodium Molybdate Dihydrate (Na_2_MoO_4_·2H_2_O), Chitosan, and TNZ were purchased from Shanghai Aladdin Biochemical Technology Co., Ltd. Disodium Hydrogen Phosphate Dodecahydrate (Na_2_HPO_4_·12H_2_O) and Sodium Dihydrogen Phosphate Dihydrate (NaH_2_PO_4_·2H_2_O) were purchased from Tianjin Lianye Chemical Reagent Co., Ltd. All chemicals used were of AR grade with a purity of 99 %. Unless otherwise specified, they were used as received without any modifications. The deionized water (DI) was used throughout this experiment.

### 2.2 Characterization

The CH650E electrochemical workstation (Shanghai Chenhua Company), Ultrasonic Cell Disruptor (Ningbo Xinzhi JY92-IIDN), IRAffinity-1 Fourier Transform Infrared (FTIR) Spectrometer, AXIS Nova X-ray Photoelectron Spectrometer (Japan Shimadzu Corporation), HITACHI-SU8600 Scanning Electron Microscope (SEM), HITACHI-HT7800 Transmission Electron Microscope (TEM) (Japan Hitachi Corporation), XRD-6100 X-ray Diffractometer (Germany Bruker Corporation), and SXH-5-12LTP Muffle Furnace (Shanghai Yitian Scientific Instrument Co., Ltd.) have been employed for characteration.

### 2.3 Synthesis of MnMoO_4_/g-C_3_N_4_/CHIT by sonochemical method

Initially, 0.1 M MnCl_2_·4H_2_O and 0.2 M Na_2_MoO_4_·2H_2_O were well mixed, and then the resulting mixture suspension was sonicated using an Ultrasonic Cell Disruptor (with a sound power of 500 W and frequency of 30 kHz) for 15 min at room temperature (25 °C) to prepare the MnMoO_4_ suspension [26]. Subsequently, 500 mg of urea was added to the mixture. The addition of urea in this synthesis aimed at guiding the uniform growth of particles and maintaining the pH value of the reaction medium. Ultrasonic waves can induce the powerful cavitation effects in liquids, leading to the intense oscillation of the solvent and hydrodynamic effects. This aids in the mixing and dispersing of the solid particles. Therefore, the suspension was sonicated again for 1 h. After filtering, the white solution was repeatedly cleaned with ethanol and the deionized water. Ultimately, an oven was used to dry the gathered white precipitate until it was fully dried. Subsequently, it was calcined in an air atmosphere at 600° C for 3 h, resulting in a gray-black solid. This product was named MnMoO_4_ NPs.

Preparation of g-C_3_N_4_: A 50 mL covered Al_2_O_3_ crucible containing 10 g of urea was placed in a muffle furnace and heated at a rate of 4 °C per minute to 550 °C where it was maintained for 4 h. Subsequently, the sample was cooled to the room temperature, removed from the furnace, and pulverized. The yellow powder produced was identified as g-C_3_N_4_.

**Scheme 1:**
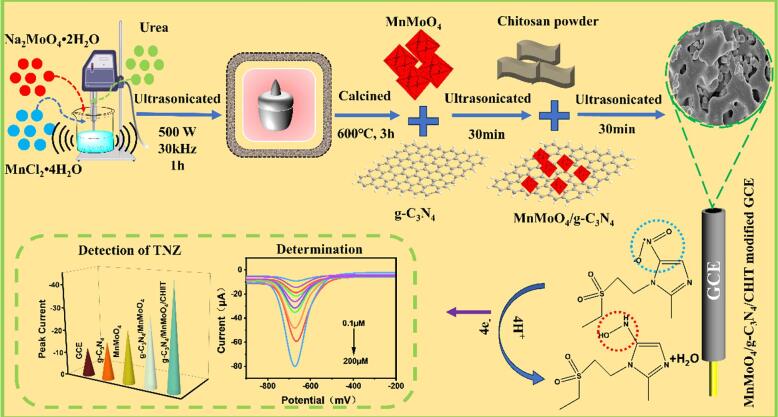
The preparation process of the MnMoO_4_/g-C_3_N_4_/CHIT/GCE electrochemical sensor.

An equal mole of g-C_3_N_4_ and MnMoO_4_ NPs were dispersed in deionized water and sonicated for 1 h. The resulting solution was subsequently dried overnight in an oven at 80 °C to yield MnMoO_4_ /g-C_3_N_4_. 30 mg of chitosan was dissolved in a 10 mL 3 % acetic acid solution [27]. Subsequently, 20 mg of MnMoO_4_ /g-C_3_N_4_ was added and homogenized by ultrasonication. The mixture was then dried in an oven to produce the product MnMoO_4_/g-C_3_N_4_/CHIT. The synthesis process is illustrated in Scheme 1.

### 2.4 Fabrication of the modified electrode

Before modification, the solid-phase polished GCEs were prepared using 1.0 μm, 0.30 μm, and 0.05 μm alumina, respectively. Afterwards, the MnMoO_4_/g-C_3_N_4_/CHIT was drop-casted onto the surface, followed by the evaporation at room temperature (MnMoO_4_/g-C_3_N_4_/CHIT) to modify GCE. The suspension was prepared by dispersed 5 mg of MnMoO_4_/g-C_3_N_4_/CHIT in 5 mL of the deionized water for the electrode modification. Using the aforementioned dispersed phase, MnMoO_4_ (MnMoO_4_/GCE), g-C_3_N_4_ (g-C_3_N_4_/GCE) and MnMoO_4_/g-C_3_N_4_ (MnMoO_4_/g-C_3_N_4_) modified GCEs were prepared following a similar procedure.

### 2.5 The electrochemical tests

A typical three-electrode setup was used for the electrochemical testing: a platinum sheet served as the counter electrode, a saturated calomel electrode (SCE) served as the reference electrode, and the working electrodes were bare GCE, g-C_3_N_4_/GCE, MnMoO_4_/GCE, or MnMoO_4_/g-C_3_N_4_/CHIT/GCE. The pH was adjusted using phosphoric acid and sodium hydroxide for acid-base regulation. The electrochemical test and the electrochemical impedance spectroscopy (EIS) were performed in a 0.1 M KCl solution containing 5.0 mM K_3_[Fe(CN)_6_]/K_4_[Fe(CN)_6_]. The electrochemical experiments were conducted in the PBS solution (0.1 M NaH_2_PO_4_-Na_2_HPO_4_, pH=5.0). Voltammetric curves were recorded using the cyclic voltammetry (CV) at a scan rate of 50 mV/s.

The parameters for differential pulse voltammetry (DPV) were presented as follows: pulse height 0.004 V, pulse amplitude 0.050 V, period 0.2 s, and potential ranging from −0.1 V to −1.0 V. The voltammetric curves shown are obtained after subtracting the background current at room temperature. 24.727 mg of TNZ standard substance was weighed and dissolved in a 100 mL volumetric flask with the deionized water and made up to volume, obtaining a 1.0 mM TNZ standard solution, which was then refrigerated. When in use, it was diluted to the desired concentration with the PBS solution.

### 2.6 Testing of real sample

The river water was sampled from the nearby Songhua River in the Harbin city, Heilongjiang province, China, while the tap water was obtained from the laboratory. The required actual samples were filtered through a 0.22 μm microporous membrane to remove the impurities from the river water. 5.0 mL of the filtered sample was then transferred into a 10 mL beaker, followed by the addition of 5.0 mL of the pre-prepared 0.1 M PBS solution (pH=5.0).

## 3 Results and discussion

### 3.1 Material characterization

**Fig. 1:**
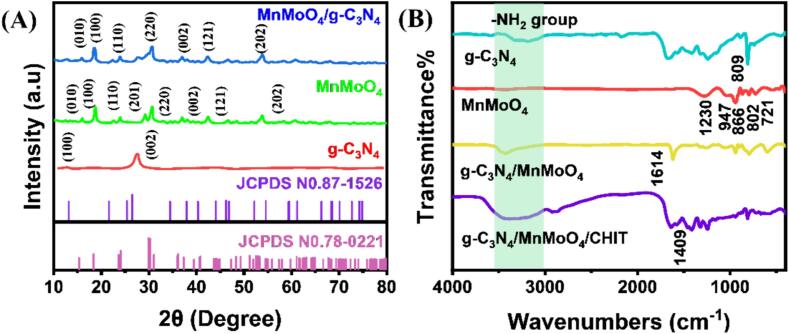
The XRD (A) and Fourier transform infrared spectra (B) of g-C_3_N_4_, MnMoO_4_, MnMoO_4_/g-C_3_N_4_ and MnMoO_4_/g-C_3_N_4_/CHIT.

Fig. 1 (A) shows the X-ray diffraction (XRD) patterns of g-C_3_N_4_, MnMoO_4_, and MnMoO_4_/g-C_3_N_4_ composite materials. The planar structure of the triazine ring units and the aromatic system's interlayer stacking structure in g-C_3_N_4_ (JCPDS No. 87–1526) are responsible for these peaks [28]. Besides the characteristic peaks of g-C_3_N_4_, the distinct diffraction peaks of MnMoO_4_ (JCPDS No.78–0221) can be also observed at 15.8°, 18.8°, 24.1°, 29.6°, 36.7°, 42.4°, and 53.7°, corresponding to the (0 1 0), (1 0 0), (1 1 0), (2 2 0), (0 0 2), (1 2 1), and (2 0 2) crystal planes, respectively [29]. FT-IR was used to further analyze the products' local structure and surface coordination, as illustrated in Fig. 1 (B). The stretching vibration of the s-triazine rings is connected to the particular absorption band of g-C_3_N_4_ at 809 cm^−1^ [30]. The absorption peaks at 1573, 1408, 1330, and 1250 cm^−1^ correspond to the aromatic C-N stretching vibration peaks. The stretching vibration of –CN can be observed by the absorption band at 1640 cm^−1^. The broad absorption band in the range of 3100 ∼ 3350 cm^−1^ corresponds to the vibration peak of the –NH_2_ functional group. The stretching vibration of Mo = O is represented by the peak at 939 cm^−1^ in the FTIR spectrum of MnMoO_4_, the bending vibration of Mo-O-Mo is represented by the peak at 897 cm^−1^, the stretching vibration of Mo-O tetrahedral MoO_4_- is represented by the peaks at 843 cm^−1^ and 798 cm^−1^, and the Mo-O vibration of MoOx is represented by the peak at 599 cm^−1^. In the FT-IR spectrum of MnMoO_4_/g-C_3_N_4_ composite material, in addition to the relevant peaks of MnMoO_4_, a strong peak can be observed at 1200 ∼ 1650 cm^−1^, matching the aromatic C-N stretching vibration in g-C_3_N_4_, indicating the successful preparation of MnMoO_4_/g-C_3_N_4_ composite material [31]. Strong evidence supporting the existence of the MnMoO_4_/g-C_3_N_4_/CHIT backbone can be seen in the FT-IR of the MnMoO_4_/g-C_3_N_4_/CHIT nanocomposite, with the peaks at 963 cm^−1^, 1081 cm^−1^, and 1409 cm^−1^ characterized as the stretching vibrations of C-O-C and C-N from the axial transformation of CHIT [32].

### 3.2 Structural and morphological studies

**Fig. 2:**
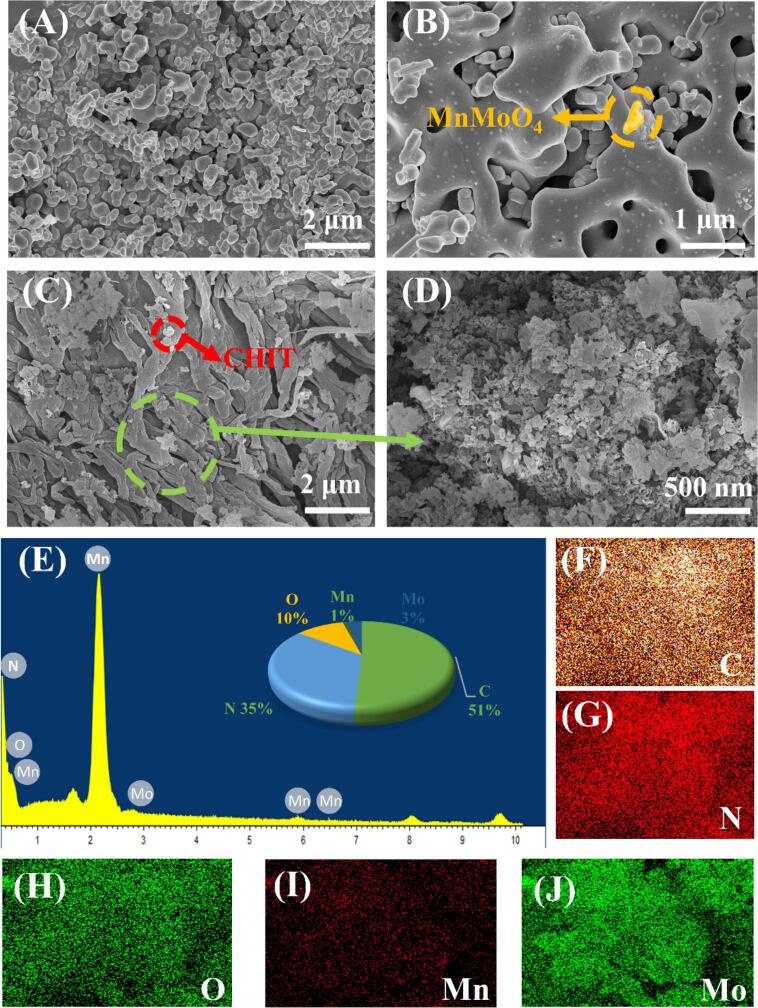
(A) The SEM image of MnMoO_4_, (B) The SEM image of MnMoO_4_/g-C_3_N_4_, (C-D) The SEM image of MnMoO_4_/g-C_3_N_4_/CHIT with different scales, (E) the EDS spectrum of MnMoO_4_/g-C_3_N_4_/CHIT, elemental mapping analysis of the MnMoO_4_/g-C_3_N_4_/CHIT (F-J).

The surface morphologies of MnMoO_4_, g-C_3_N_4_, MnMoO_4_/g-C_3_N_4_, and MnMoO_4_/g-C_3_N_4_/CHIT were investigated using scanning electron microscopy (SEM). The SEM pictures of MnMoO_4_ are displayed in Fig. 2(A), namely the surface exhibits finger-like or smooth nanostructures. Fig. 2 (B) showed the SEM image of MnMoO_4_/g-C_3_N_4_ composite material at a scale of 1 μm, revealing MnMoO_4_ nanorods embedded on g-C_3_N_4_. This indicated that the formation of MnMoO_4_ nanoparticles on the g-C_3_N_4_ the sheets. Fig. 2 (C-D) showed the SEM images of MnMoO_4_/g-C_3_N_4_/CHIT at different magnifications. According to the SEM images, CHIT aggregates on the MnMoO_4_/g-C_3_N_4_ nanoparticles, demonstrating the successful preparation of MnMoO_4_/g-C_3_N_4_/CHIT. In addition, SEM characterization reveals that the surface of the composite material becomes smooth after the addition of chitosan, with the MnMoO_4_ particles aggregating to form the clustered structures. The high-magnification SEM image depicted in Fig. 2 (D) describes CHIT as a flexible thin film with numerous wrinkles. More importantly, MnMoO_4_/g-C_3_N_4_ is found to be tightly wrapped by CHIT, forming a conductive network that promotes the rapid electron transfer. As seen in Fig. 2 (E-J), the EDS analysis further verifies the existence of components including C, N, O, Mn, and Mo in the MnMoO_4_/g-C_3_N_4_/CHI nanocomposites.

**Fig. 3:**
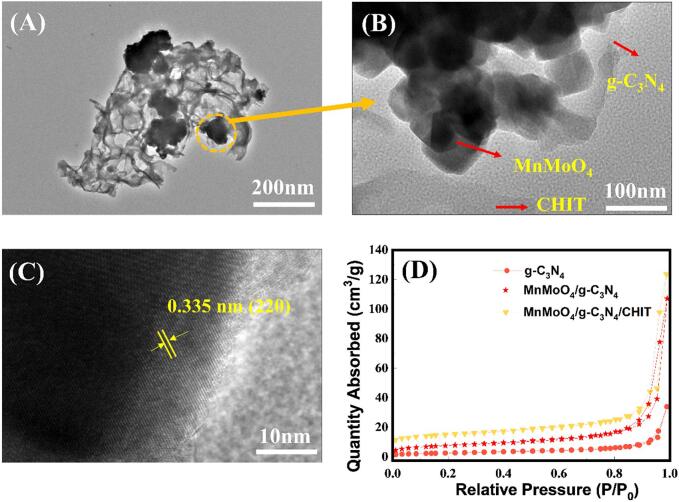
The TEM Analysis of MnMoO_4_/g-C_3_N_4_/CHIT hybrid material. Low-resolution image (a), High-resolution images (b and c), and the BET testing for g-C_3_N_4_ and MnMoO_4_/g-C_3_N_4_/CHIT (d).

We further investigated the microstructure of MnMoO_4_/g-C_3_N_4_/CHIT hybrid composite using the TEM characterization (Fig. 3a-c). The TEM images of the hybrid exhibit a three-dimensional structure where one-dimensional CHIT intertwines with the g-C_3_N_4_ layers. Moreover, the coexisting MnMoO_4_ rods are tightly interconnected with the conductive g-C_3_N_4_/CHIT three-dimensional network. The resulting ternary composite is expected to possess high charge carrier characteristics and excellent ion storage capability, thereby demonstrating the enhanced adsorption ability for the detection of tinidazole. Furthermore, HR-TEM reveals a lattice spacing of 0.335 nm corresponding to the (2 2 0) plane (Fig. 3c), which agree swell with the XRD analysis.

The BET testing is commonly used to characterize the specific surface area of the electrode materials. BET testing can help determine their specific surface areas, which is crucial to understand their potential applications in adsorption, catalysis, and other areas [33]. Based on the pore size distribution experiments, it was found that MnMoO_4_/g-C_3_N_4_/CHIT has a greater number of pores compared with the unmodified g-C_3_N_4_. This further suggests that MnMoO_4_/g-C_3_N_4_/CHIT possesses more binding sites and a faster rate of electron transfer, implying that MnMoO_4_/g-C_3_N_4_/CHIT can facilitate the rapid and efficient electron transfer in the electrochemical reactions, thereby promoting the progress of the reactions. The specific surface area, pore volume, and average pore size of g-C_3_N_4_, MnMoO_4_/g-C_3_N_4_, and MnMoO_4_/g-C_3_N_4_/CHIT were calculated and are shown in Table S1. It is evident from the table's statistics that MnMoO_4_/g-C_3_N_4_ shows the increased specific surface area, pore volume, and the average pore size following modification. Moreover, the average pore size, pore volume, and specific surface area are all increased by the addition of CHIT. This may attribute to the effect of ultrasound, which could induce the formation of the surface irregularities on the metal, thereby increasing the surface roughness， and consequently further enhancing the effective surface area of the material. Furthermore, under the influence of ultrasound, more surface areas that were originally obscured can be retained, further augmenting the material's surface area. These results demonstrate that the prepared MnMoO_4_/g-C_3_N_4_/CHIT possesses a larger surface area, facilitating the aggregation of TNZ, thereby leading to a lower limit of detection for TNZ.

## 4 Electrochemical performances

### 4.1 Electrochemical performance of different electrodes

**Fig. 4:**
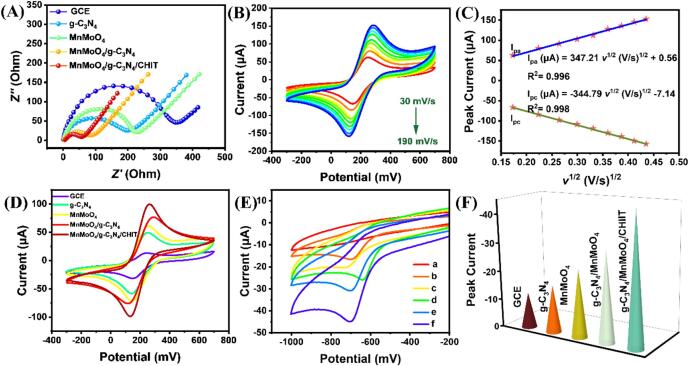
(A) The electrochemical impedance spectroscopy (EIS) plots of different electrodes in [Fe(CN)_6_]^3–/4–^ solution; (B) the CV curves of MnMoO_4_/g-C_3_N_4_/CHIT/GCE at different scan rates (30 ∼ 190 mV/s) in [Fe(CN)_6_]^3–/4–^ solution; (C) the square root of scan rate (V/s)^1/2^ and peak current (μA) have a linear connection; (D) the CV curves for various electrode types; (E) the CV plots in PBS (0.1 M, pH=5.0) without TNZ and with 100.0 μM TNZ (a: MnMoO_4_/g-C_3_N_4_/CHIT/GCE; b: GCE+TNZ; c: g-C_3_N_4_/GCE+TNZ; d: MnMoO_4_ /GCE+TNZ; e: MnMoO_4_/g-C_3_N_4_/GCE+TNZ; f: MnMoO_4_/g-C_3_N_4_/CHIT+TNZ); (F) the corresponding current magnitudes.

Through electrochemical impedance spectroscopy (EIS), the response patterns of different nanomaterial-modified electrodes, including MnMoO_4_/g-C_3_N_4_/CHIT/GCE, MnMoO_4_/g-C_3_N_4_/GCE, MnMoO_4_/CPE, and unmodified GCE, were evaluated, as shown in Fig. 4 (A). The diameter of the semicircle in the high-frequency region of the EIS spectra represents the electrode's charge transfer resistance (Rct), whereas the electrode's diffusion effects are mostly reflected in the linear part in the low-frequency zone [34], [35]. According to Fig. 4 (A), the GCE's Rct is 335.1 Ω. After modification with MnMoO_4_ and g-C_3_N_4_, Rct significantly decreases, indicating the accelerated electron transfer at the electrode modified by MnMoO_4_ and g-C_3_N_4_. The Rct of MnMoO_4_/g-C_3_N_4_/GCE is even smaller (79.1 Ω), suggesting that MnMoO_4_/g-C_3_N_4_ facilitates electron transfer more effectively. When CHIT is incorporated into MnMoO_4_/g-C_3_N_4_, the Rct of MnMoO_4_/g-C_3_N_4_/CHIT/GCE decreases to 62.6 Ω. Considering the SEM images of MnMoO_4_/g-C_3_N_4_/CHIT, it is possible that CHIT increases the specific surface area of the nanomaterial and provides more active sites for electron transfer.

The cyclic voltammetry (CV) curves obtained by scanning at different rates with MnMoO_4_/g-C_3_N_4_/GCE in a 5 mM [Fe(CN)6]^3−/4−^ solution containing 0.1 M KCl are shown in Fig. 4(B). The cyclic voltammetry (CV) curves obtained by scanning at different rates with GCE in a 5 mmol/L [Fe(CN)6]^3−/4−^ solution containing 0.1 mol/L KCl are shown in Fig. S1(A-B). When the scan rate increased from 30 mV/s to 190 mV/s, the square root of the scan rate (*v*^1/2^) and the peak current for reduction showed a decent linear relationship (R^2^_pa1_ = 0.996, R^2^_pa2_ = 0.998) (Fig. 4C). This indicated that the electrocatalytic reaction of [Fe(CN)_6_]^3−/4−^ on MnMoO_4_/g-C_3_N_4_/CHIT/GCE is a diffusion-controlled process. The electrochemically active surface area (A) of the electrode was calculated using the Randles-Sevcik equation according to equation (1)[36], [37]:

(1) $${\text{I}}_{\text{pa}}\text{=2.69}\times {\text{10}}^{\text{5}}\text{A}{\text{D}}^{\text{1/2}}{\text{n}}^{\text{2/3}}{\text{v}}^{\text{1/2}}\text{C}$$

Among them, n is the number of electrons, D is the diffusion coefficient of [Fe(CN)_6_]^3-/4-^ in the solution (6.7 × 10^-6^ cm^2^/s). C is the concentration of [Fe(CN)_6_]^3-/4-^ (5 × 10^-6^ mol/cm^3^), *v*^1/2^ is the square root of the scan rate, and I_pa_ is the anodic peak current.

The electrochemical active surface areas of MnMoO_4_/g-C_3_N_4_/CHIT/GCE and bare GCE are around 0.1081 cm^2^ and 0.0724 cm^2^, respectively, according to calculations. This indicates that MnMoO_4_/g-C_3_N_4_/CHIT/GCE possesses a higher electrochemical active surface area, which is beneficial for the abundant accumulation of TNZ on the electrode surface, thereby enhancing the current response. Fig. 4(D) further illustrates the current response values of different modified electrodes in [Fe(CN)_6_]^3-/4-^ solution presented as follows: MnMoO_4_/g-C_3_N_4_/CHIT/GCE>MnMoO_4_/g-C_3_N_4_/GCE>MnMoO_4_/GCE>g-C_3_N_4_/GCE>GCE. MnMoO_4_/g-C_3_N_4_/CHIT/GCE exhibits the strongest current response, which can be explained by the high specific surface area of g-C_3_N_4_ and the synergistic effect of binary transition metal oxides on the energy barriers in the electrocatalytic processes, which improves the material's electrical conductivity and the catalytic activity, and the protonation of –OH and –NH_2_ in chitosan to form cationic polymers [38]. In addition, the glucose units in chitosan molecules have a degree of conjugated structure, which aids in the electron conduction. Numerous nitrogen functional groups, including amines and nitrides, can offer active sites and catalytic activity in g-C_3_N_4_. Consequently, the collaborative efforts of these materials can lead to a substantial dispersion [39].

### 4.2 The electrochemical response of MnMoO_4_/g-C_3_N_4_/CHIT to TNZ

To demonstrate the performance of the MnMoO_4_/g-C_3_N_4_/CHIT composite in the TNZ detection, five different electrodes were compared under the same conditions, including GCE, g-C_3_N_4_/GCE, MnMoO_4_/GCE, MnMoO_4_/g-C_3_N_4_/GCE, and MnMoO_4_/g-C_3_N_4_/CHIT, as shown in Fig. 4 (E-F). Using cyclic voltammetry (CV) within the potential range of −1.0 to −0.2 V at a scan rate of 50 mV/s, the electrochemical behavior of 100 μm TNZ in 0.1 M PBS (pH=5) was studied with different electrodes. In the blank PBS solution, no CV signal of the target analyte was observed for MnMoO_4_/g-C_3_N_4_/CHIT, as shown in Fig. 4 (E). TNZ on the modified electrode surface exhibited no oxidation peak during the reverse scan, indicating that the electrochemical reduction of TNZ on the modified electrode surface is an irreversible reaction. Moreover, the reduction peak current observed for TNZ on the pristine glassy carbon electrode (GCE) surface exhibits a subdued intensity, indicating the subdued electron transfer kinetics of TNZ on the pristine GCE. After modification, the electrode exhibited a significant enhancement in the current response, along with a shift in the peak potential towards more negative values as compared to the unmodified GCE. The differences in the peak potential among different electrodes for TNZ could be attributed to the differences in electrode materials and supporting electrolytes. In addition, the modified electrode exhibited more pronounced reduction peaks, which may be attributed to the higher oxidation states of molybdenum (Mo) and manganese (Mn) in MnMoO_4_ [40], [41]. Under the high oxidation states, the ions of molybdenum and manganese exhibit decent electrochemical activity. Moreover, MnMoO_4_ possesses a unique crystal structure and surface morphology, which might offer a greater specific surface area and more active spots, thereby increasing the efficiency of the reduction reaction [42]. Chitosan, on the other hand, exhibits excellent ion exchange properties, which can promote electron transfer and improve electrode conductivity. These results suggest a significant improvement in the TNZ detection with MnMoO_4_/g-C_3_N_4_/CHIT.

### 4.3 Optimization of experimental conditions

#### 4.3.1 Effect of the pH value

**Fig. 5:**
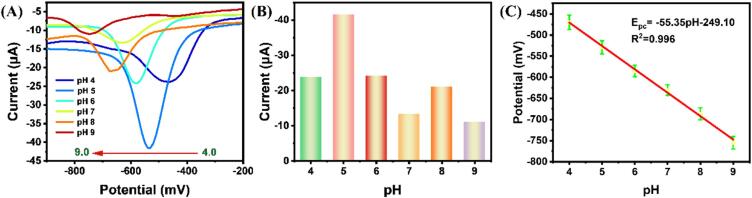
(A) The DPV of 100 µM TNZ at different pH values of 0.1 M PBS at a scan rate of 50 mV/s; (B) the current at different pH values; (C) the relationship between the pH value and voltage.

The effect of pH value in electrolyte solution (100 µM TNZ) on the electrochemical response was investigated using differential pulse voltammetry (DPV) with a scan rate of 50 mV/s, within the pH range of 4.0–9.0 as shown in Fig. 5 (A). As depicted in Fig. 5 (B-C), within the pH range from 4.0 to 5.0, the cathodic current response of TNZ was found to be sharply increased, but further increases in the pH value led to a reduction in the TNZ current response. As a result, pH 5.0 was determined to be the optimal level for further research. The adverse reactions at lower pH value can be attributed to the proton consumption involvement in the electrode process. As the pH increases, the cathodic current response decreases, indicating the potential proton deficiency or the possibility of analyte deprotonation at higher pH values, transitioning from cations to anions. The individual analytes and the changed electrode may get attracted to one another electrostatically as a result of this transition, perhaps lowering the current response. Based on Fig. 4 (C), the relationship between pH and peak potential is described by the equation: E_pc_ = -55.55 pH − 249.10 (R^2^ = 0.996). The slope values of the equation are close to −59 mV/pH. This demonstrated that in the electrochemical reduction process of TNZ, the number of electrons is equal to the number of protons [43]. Therefore, with the pH values increases, the cathodic potential of TNZ shifts towards more negative values. This change in the analyte's pH value might be the result of an equal amount of the proton and electron transfers, which would affect the overall electrochemical behavior.

#### 4.3.2 Influence of different amount of catalyst loading on GCE

The loading level of the catalyst (MnMoO_4_/g-C_3_N_4_/CHIT nanocomposite) on the surface of the GCE is a critical parameter for the efficient detection of the target analyte (TNZ). To explore the optimization of loading levels, varying volumes (4, 6, 8, and 10 mL) were extracted from the uniformly prepared suspension and subsequently drop-cast onto pre-cleaned GCE surfaces. Each independently prepared electrode (4 mL − MnMoO_4_/g-C_3_N_4_/CHIT/GCE, 6 mL − MnMoO_4_/g-C_3_N_4_/CHIT/GCE, 8 mL − MnMoO_4_/g-C_3_N_4_/CHIT/GCE, and 10 mL- MnMoO_4_/g-C_3_N_4_/CHIT/GCE) was studied at a constant scan rate of 50 mV/s (containing 100 mM TNZ). The obtained CV responses at different catalyst loading volumes are shown in Fig. 5(A). It is evident that as the catalyst loading volume increases from 4 mL to 6 mL, the cathodic CV signal of TNZ also increases; however, as the loading amount increases from 6 mL to 10 mL, the reduction current response of TNZ decreases. This may be attributed to the aggregation of MnMoO_4_/g-C_3_N_4_/CHIT composite material, leading to the hindered electron transfer of surface modifiers. In summary, 6 mL of MnMoO_4_/g-C_3_N_4_/CHIT/GCE is chosen as the optimal loading volume for further electrochemical experiments.

#### 4.3.3 Effect of scan rate

**Fig. 6:**
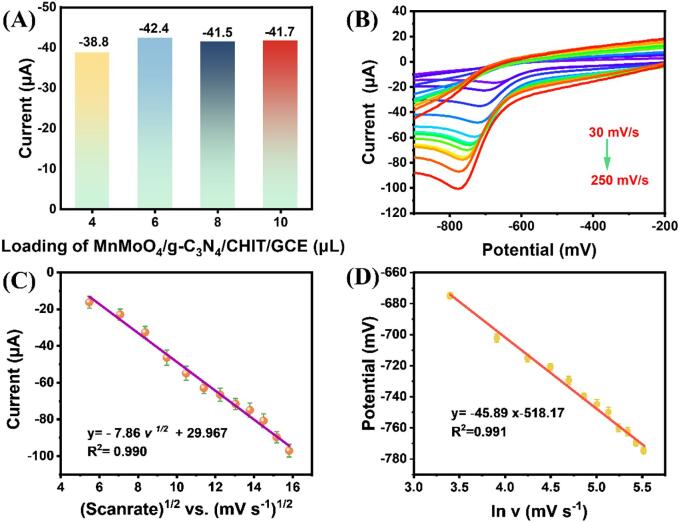
(A) The bar chart obtained from CV results (cathodic current (mA) vs. the load of MnMoO_4_/g-C_3_N_4_/CHIT (mL)) for different catalyst loadings (4, 6, 8, and 10 mL); (B) The CV curves obtained at the scan rates of 30 to 250 mV/s with a scan interval of 20 mV/s; (C) The linear calibration plot between the square root of scan rate (mV/s) and current (mA); (D) The plot demonstrating linear calibration between the cathodic potential (Epc/V) and the natural logarithm of the scan rate (ln mV s^−1^).

In a 0.1 M PBS solution containing 100 μM TNZ, the effect of scan rate on cyclic voltammetry (CV) was investigated within the range of 30–250 mV s^−1^. As shown in Fig. 6 (B), with the increase in the scan rate from 30 to 250 mV s^−1^, the peak current gradually increases. Furthermore, the peak potential shifts towards more negative values, indicating an irreversible electrochemical reduction process. The peak current is linearly related to the square root of the scan rate, with a regression equation of I_pc_(µA) = − 7.86 *v*^1/2^ (mv ^-1^)^1/2^ ––2.68, R^2^ = 0.990 (Fig. 6 C). The irreversible reduction of TNZ on MnMoO_4_/g-C_3_N4/CHIT/GCE appears to be diffusion-controlled, as evidenced by the cathodic peak current (Ipc) of TNZ increasing linearly with the square root of the scan rate [44].

Concurrently, there is a negative shift in the peak potential (Epc) of TNZ when the scan rate varies between 30 and 250 mV/s. The relationship between Epc and the natural logarithm of the scan rate (ln *v*) follows the equation: E_pc_ = -45.89 ln *v* −518.87 (R^2^ = 0.991). The slope of the Laviron equation may be used to calculate the number of transferred electrons (n) during the electrochemical process [27].

(2) $$E_{\mathrm{PC}}=E^{0}+\frac{\mathrm{RT}}{\alpha nF}\ln\frac{\mathrm{RTKs}}{\alpha nF}-\frac{\mathrm{RT}}{\alpha nF}$$

Assuming *α* to be 0.5 and the value of n for TNZ to be 1.49, the results indicate that the rate-determining step of the electrochemical reaction involves the participation of one electron. According to references [45], [46], there are two phases involved in the electrochemical reduction of TNZ. Four protons and four electrons participate in the complete electrochemical reduction process from –NO_2_ to –NHOH, with the slow reaction (involving one electron) serving as the rate-determining step (Scheme S1). The possible electrochemical reduction mechanism of TNZ on the MnMoO_4_/g-C_3_N_4_/CHIT/GCE electrode can be seen in Scheme S2.

#### 4.3.4 Quantitative determination of TNZ on MnMoO_4_/g-C_3_N_4_/CHIT/GCE

**Fig. 7:**
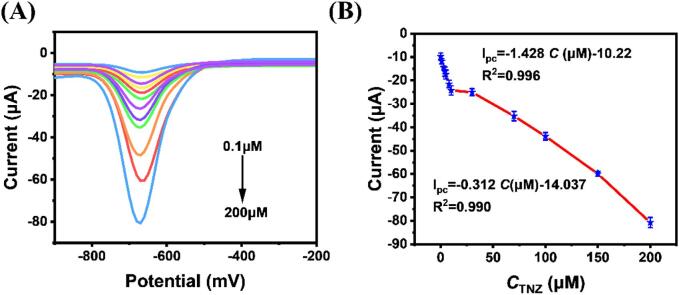
The DPV curves of the MnMoO_4_/g-C_3_N_4_/CHIT/GCE in the presence of different concentrations of TNZ in 0.1 M PBS solution (pH=5.0) (A); the calibration plot of reduction peak current versus TNZ concentration (B).

Using the differential pulse voltammetry (DPV) technique, different concentrations of TNZ were determined on MnMoO_4_/g-C_3_N_4_/CHIT in 0.1 M PBS (pH=5.0). The DPV curve (Fig. 7A) demonstrates a linear increase in the reduction peak current with the gradual addition of TNZ (0.1 ∼ 200 μM). Fig. 7(B) showed the association between the concentration and the TNZ current signal. Two linear equations can be obtained for TNZ within its linear range. The corresponding equations are as follows: Ipc = -1.428*C* (μM)-10.22 (R^2^ = 0.996), Ipc = -0.312*C* (μM)-14.027 (R^2^ = 0.990).

**Scheme 2:**
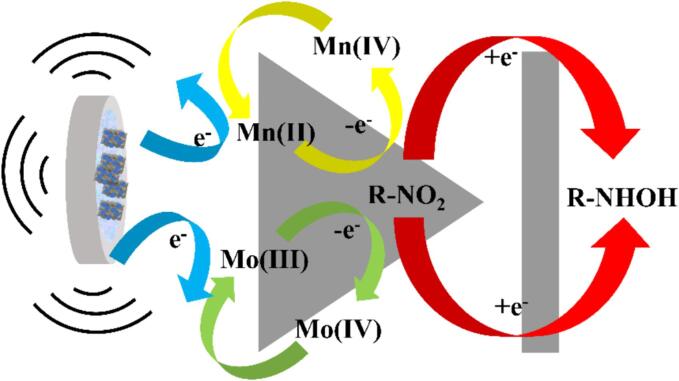
The contributions of MnMoO_4_ species to the electrochemical reduction of TNZ.

The formula for the detection limit (LOD) is 3*SD/N, where N is the calibration curve's slope and SD is the standard deviation of the blank samples [46]. The LOD is determined to be 3.78 nM. In addition, a sensitivity of 1.320 µA·µM^−1^·cm^−2^ can be evaluated. Table 1 presents a comparison between MnMoO_4_/g-C_3_N_4_/CHIT/GCE and a few other previously published sensors for the electrochemical detection of TNZ. The research findings indicate that the MnMoO_4_/g-C_3_N_4_/CHIT/GCE sensor demonstrates satisfactory sensing performance in detecting tinidazole (TNZ). Although ChCl/GCE has a lower LOD and a wider detection range for TNZ when compared with MnMoO_4_/g-C_3_N_4_/CHIT/GCE, the preparation process of MnMoO_4_/g-C_3_N_4_/CHIT/GCE is relatively simple and does not produce pollutants, resulting in the minimal environmental impact. The excellent electrocatalytic capability of the MnMoO_4_/g-C_3_N_4_/CHIT/GCE towards TNZ can be attributed to several reasons. Firstly, the MnMoO_4_/g-C_3_N_4_ composite's interconnected porous structure makes it easier for TNZ to enrich on the electrode surface, which improves the response current. Secondly, the addition of CHIT improved the conductivity of the material, while ultrasound reduced the tendency of MnMoO_4_/g-C_3_N_4_ nanoparticles to aggregate, promoting better interaction between the catalytic sites and TNZ. Lastly, the MnMoO_4_ in MnMoO_4_/g-C_3_N_4_ possesses strong reducibility, which can promote the electrochemical reduction of TNZ, resulting in higher peak currents. Specifically, the –NO_2_ group of TNZ easily interacts with the active centers Mn (II) and Mo (III), and then undergoes a four-electron process to be reduced to –NHOH. During the –NO_2_ –NHOH electroreduction process, the low-valent state of Mn (II)/Mo (III) may convert to the the high-valent state of Mn(IV)/Mo(IV)/Mo(VI). Then the electrochemical reduction of the Mn (IV)/Mo(IV)/Mo species on the electrode surface results in Mn(II)/Mo(III). An essential component of the electrochemical reduction of TNZ is Mn(II)/Mo(III) (Scheme 2). At the same time, due to ultrasonic waves being able to generate the intense hydrodynamic effects and the localized conditions of high temperature and pressure, they reduce the activation energy of reactions and accelerate the reaction rates. This may expedite the transfer of Mn ions and Mo ions on the electrode surface, rendering MnMoO_4_/g-C_3_N_4_/CHIT/GCE highly efficient for TNZ detection.

**Table 1:** Comparison of the TNZ electrochemical study performed using various electrode configurations.

Methods	Electrodes	Linear range	LOD	Ref.
i-t	Ag-Co_3_O_4_ NPs/GCE	0.5–388.8 μM	0.035 μM	[16]
DPV	GO-chitosan/GCE	10 nM − 100 mM	3.2 nM	[27]
DPV	SG/CPE	1.0 ​μM-10.0 ​μM	0.24 μM	[45]
DPV	Ag@ZrO_2_ /GCE	0.2–414.5 μM	0.073 μM	[46]
LSV	MnO_2_/ErGO	0.1–20 μM	0.33 μM	[47]
DPV	Fe-MOF/Pt NPs	0.0196–524.956 μM	43 nM	[48]
DPV	ChCl/GCE	0.010–170 μM	0.90 nM	[49]
DPV	MnMoO_4_/g-C_3_N_4_/CHIT/GCE	0.1–200 μM	3.78 nM	this work

#### 4.3.5 Reproducibility, stability, anti-interference and selectivity ability

**Fig. 8:**
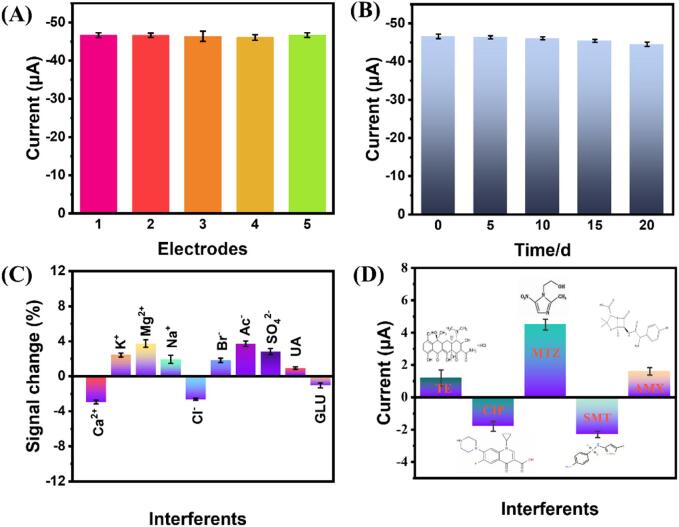
The five parallelled MnMoO_4_/g-C_3_N_4_/CHIT/GCE electrodes' current responses were measured to determine 100 μM TNZ (A); MnMoO_4_/g-C_3_N_4_/CHIT/GCE stability for the the corresponding detection of 100 μM TNZ after 20 days (B); In the presence of potential interfering substances, the current ratio of TNZ to the interferent (C-D).

In order to evaluate the reproducibility of the MnMoO_4_/g-C_3_N_4_/CHIT/GCE electrode, 100 μM TNZ was detected using five identical electrodes. It can be observed that only minor differences in current were observed among these tests in Fig. 8(A), and the DPV of these parallelled experiments are shown in Fig. S2(A).

Therefore, it can be concluded that the MnMoO_4_/g-C_3_N_4_/CHIT/GCE sensor exhibits high reproducibility. The peak current of 100 μM TNZ on the MnMoO_4_/g-C_3_N_4_/CHIT/GCE was measured every week over a period of 20 days to evaluate its stability. The results showed that TNZ could still maintain over 95 % of the original peak current after 20 days, indicating the excellent stability of the MnMoO_4_/g-C_3_N_4_/CHIT/GCE, as shown in Fig. 8(B) and Fig. S2(B). The interference resistance of the MnMoO_4_/g-C_3_N_4_/CHIT/GCE was investigated by measuring the current of TNZ (100 μM) in the presence of different interfering substances. As shown in Fig. 8(C), the addition of 50 times the concentration of Ca^2+^, K^+^, Mg^2+^, Na^+^, Cl^-^, Br^-^, uric acid, and glucose did not cause significant changes in the current of TNZ, indicating that the MnMoO_4_/g-C_3_N_4_/CHIT/GCE has decent anti-interference performance. To further validate the selectivity capability of the MnMoO_4_/g-C_3_N_4_/CHIT/GCE, 10 times the concentration of tetracycline (TE), ciprofloxacin (CIP), metronidazole (MTZ), sulfamethoxazole (SMT), and amoxicillin (AMX) were added in 0.1 M PBS solution (pH=5), as shown in Fig. 8D. MnMoO_4_/g-C_3_N_4_/CHIT exhibited a higher current response to metronidazole as compared to other antibiotics. This similarity can be attributed to the structural resemblance between metronidazole and tinidazole, as they are both nitroimidazole drugs. However, the current response of MnMoO_4_/g-C_3_N_4_/CHIT to metronidazole was lower than 6 μA, indicating its anti-interference capability under different antibiotic conditions.

#### 4.3.6 Application in real water samples

To verify the practical application potential of MnMoO_4_/g-C_3_N_4_/CHIT/GCE, samples were obtained from river water and tap water. Prior to analysis, the materials underwent pre-filtration using a 0.5 μm membrane and were then exposed to a 60-minute UV digester digestion process aimed at converting organic compounds into their inorganic forms. After diluting the sample 10 times and adding 0.1 M PBS solution (pH=5), the analysis results were obtained using the standard addition method under optimized conditions. The resulting DPV measurements are shown in Fig. S3 (A-B). As shown in Table 2, the recoveries of the river water and tap river were between 94.0 % and 106.6 %, and from 93.0 % to 102.5 % with the RSDs ranging from 1.8 % to 3.0 % and 1.2 % to 3.2 %. This indicates that the electrochemical sensor developed for this work has high accuracy and is useful for determining TNZ in the real water samples.

**Table 2:** The determination of TNZ in river water and tap water.

Samples	Added (μM)	Found (μM)	Recovery (%)	RSD (%) (n = 3)
River water	10	9.87	98.7	2.3
	30	31.98	106.6	1.8
	50	46.98	94.0	3.0
Tap water	10	9.53	95.3	1.2
	30	27.89	93.0	1.4
	50	51.23	102.5	3.2

To further verify the detection reliability and accuracy of the MnMoO_4_/g-C_3_N_4_/CHIT/GCE electrochemical sensor for TNZ in water, we assessed the consistency of the proposed MnMoO_4_/g-C_3_N_4_/CHIT /GCE sensor with the results obtained using the High-Performance Liquid Chromatography (HPLC) method. Table S2 displays the HPLC detection data for TNZ in water. The MnMoO_4_/g-C_3_N_4_/CHIT/GCE sensor's recovery rate was shown to be highly correlated with the HPLC method. The statistical analysis using a *t*-test showed no significant difference (P>0.05) between the electrochemical analysis conducted using the MnMoO_4_/g-C_3_N_4_/CHIT/GCE electrode and the HPLC method. These findings strongly indicate the effectiveness of the developed sensor for TNZ detection in water.

## 5 Conclusion

The effective synthesis of MnMoO_4_/g-C_3_N_4_/CHIT was accomplished using sonochemical methods. This composite underwent comprehensive characterization employing various techniques, uncovering its distinctive properties. Acting as a bimetallic oxide, the composite material exhibited exceptional performance in the electrochemical detection of trace tinidazole. MnMoO_4_/g-C_3_N_4_/CHIT exhibited high porosity and abundant active metal sites, effectively facilitating the electrochemical reduction and amplifying the electrochemical signal. These attributes enabled the MnMoO_4_/g-C_3_N_4_ nanocomposite to achieve a lower limit of detection (3.78 nM), a broad linear range (0.1–200 μM), high sensitivity (1.320 μA·μM^−1^·cm^−2^), exceptional selectivity, as well as decent stability in the tinidazole detection. MnMoO_4_′s robust reduction capability facilitated the electrochemical reduction of TNZ, resulting in the increased peak currents. In comparison to previous studies and references, the improved sensor exhibited a lowered detection limit, an expanded linear range, and enhanced environmental friendliness in the preparation process.

## CRediT authorship contribution statement

**Chaojun Zhang:** Writing – original draft, Formal analysis. **Rui Liu:** Writing – review & editing, Funding acquisition, Data curation. **Rijia Liu:** Methodology, Investigation. **Wenyu Cui:** Validation, Software. **Yuan Sun:** Writing – review & editing, Supervision, Funding acquisition, Conceptualization. **Wein-Duo Yang:** Investigation, Formal analysis.

## Declaration of competing interest

The authors declare that they have no known competing financial interests or personal relationships that could have appeared to influence the work reported in this paper.
