# Supplementary material for: Ultrasonically assisted fabrication of electrochemical platform for tinidazole detection
Source: Ultrason Sonochem. 2024 Sep 1;110:107056. doi: 10.1016/j.ultsonch.2024.107056 (PMC11403520; doi:10.1016/j.ultsonch.2024.107056)
Supplement: Supplementary Data 1 [file mmc1.docx]

Sonochemical fabrication of chitosan modified MnMoO_4_/g-C_3_N_4_ as an efficient sensor platform for sensitive electrochemical detection of tinidazole in water

Chaojun Zhang ^a^, Rui Liu ^a*^, Rijia Liu ^a^, Wenyu Cui ^b^, Yuan Sun ^a*^, Wein-Duo Yang ^c^

^a^ Center of Pharmaceutical Engineering and Technology, Harbin University of Commerce, Harbin 150076, China

^b^ School of Pharmacy, Harbin University of Commerce, Harbin 150076, China

^c^ Department of Chemical and Materials Engineering, National Kaohsiung University of Science and Technology, Kaohsiung 80778, Taiwan

* The corresponding authors: E-mail address: [liur@hrbcu.edu.cn;](mailto:liur@hrbcu.edu.cn;) [sunyuan.2010@163.com](mailto:sunyuan.2010@163.com;)

**Figures**

**
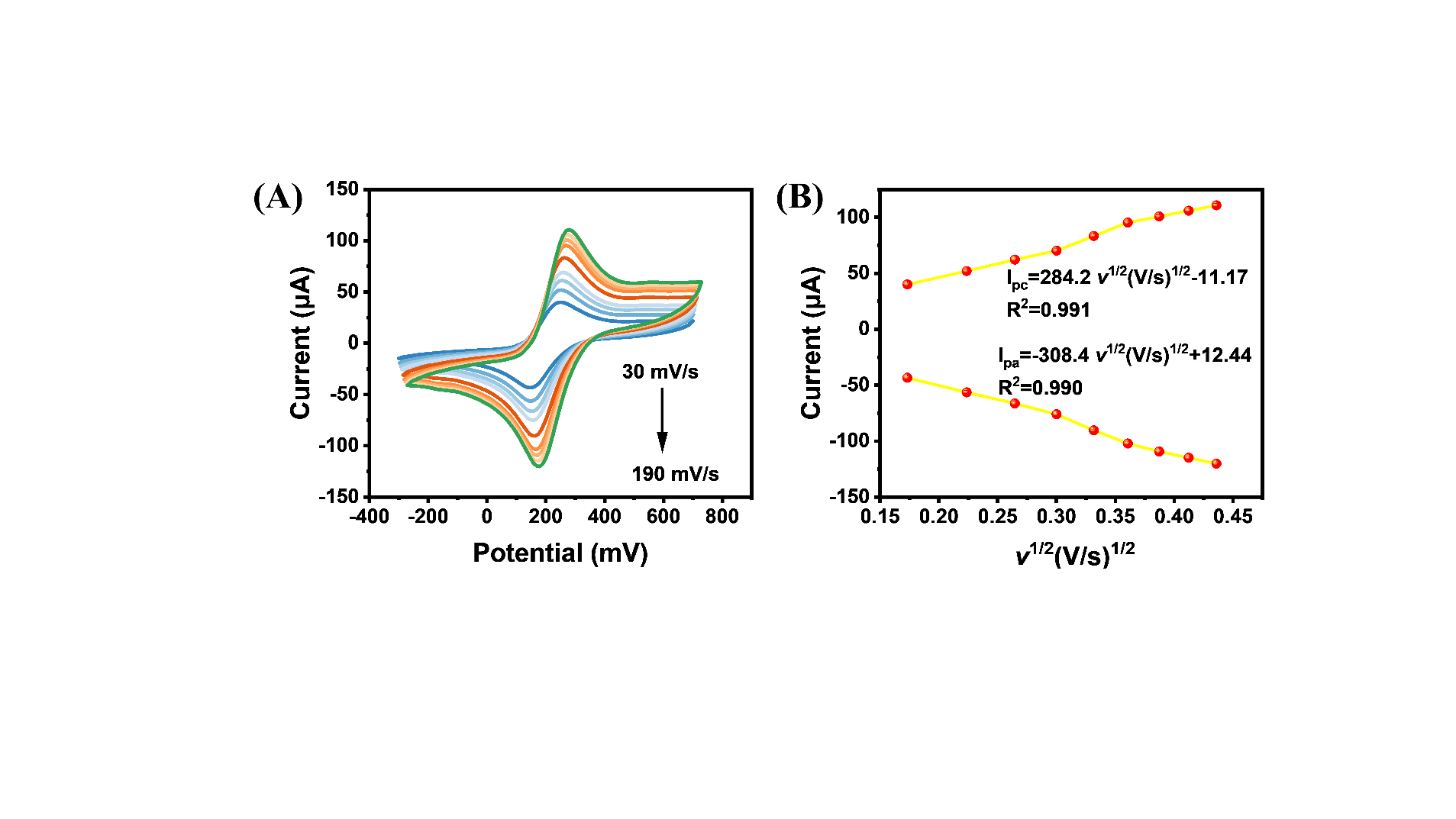
**

**FigS1**. (A) CV curves of GCE at different scan rates (30~190 mV/s) in [Fe(CN)_6_]^3–/4–^ solution; (B) Linear relationship between the square root of scan rate (V/s)^1/2^ and peak current (μA).


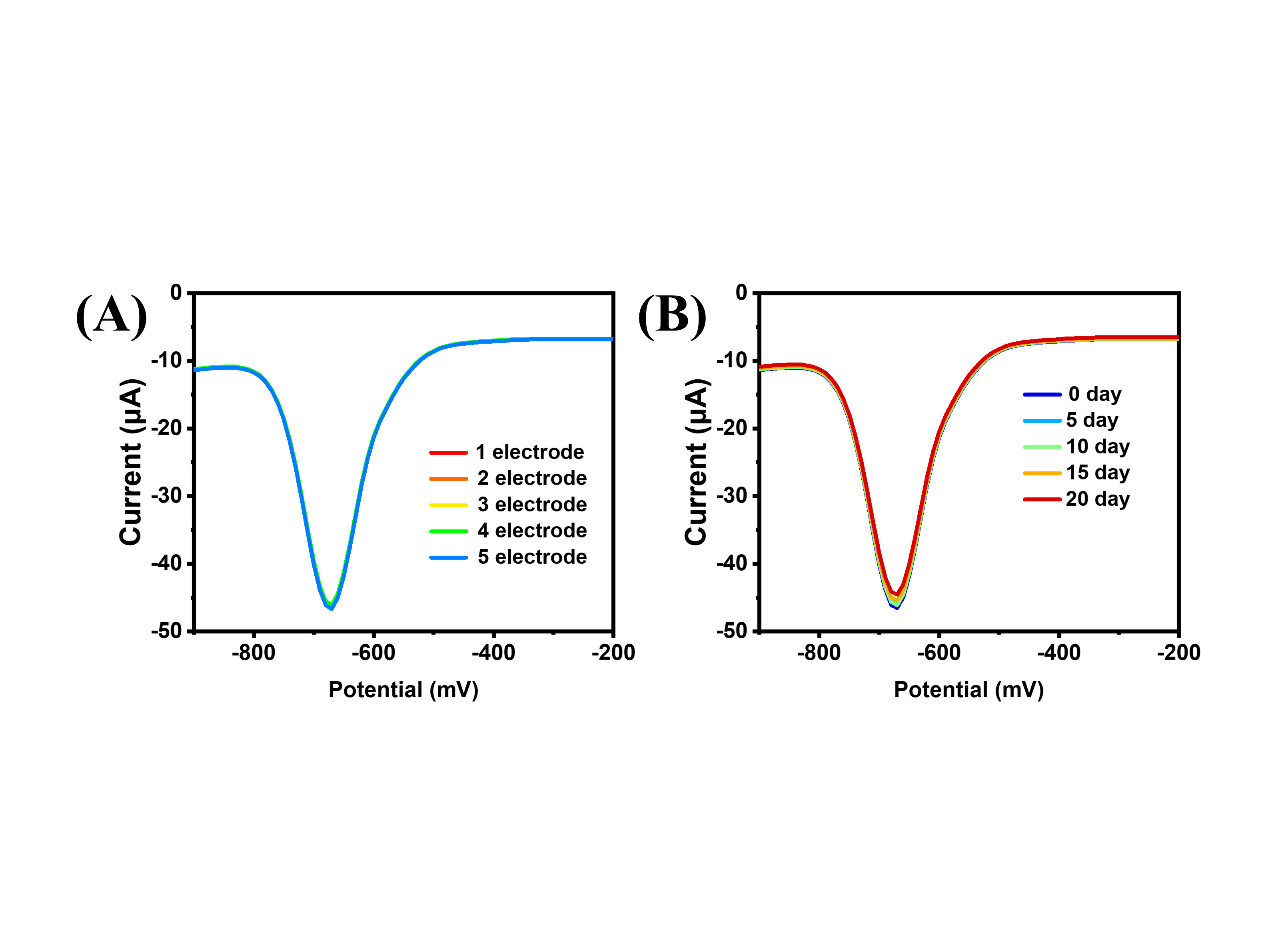


**FigS2**. (A). Differential Pulse Voltammetry (DPV) measurements of five parallel MnMoO_4_/g-C_3_N_4_/CHIT/GCE electrodes; (B) Current DPV of MnMoO_4_/g-C_3_N_4_/CHIT/GCE over different days.


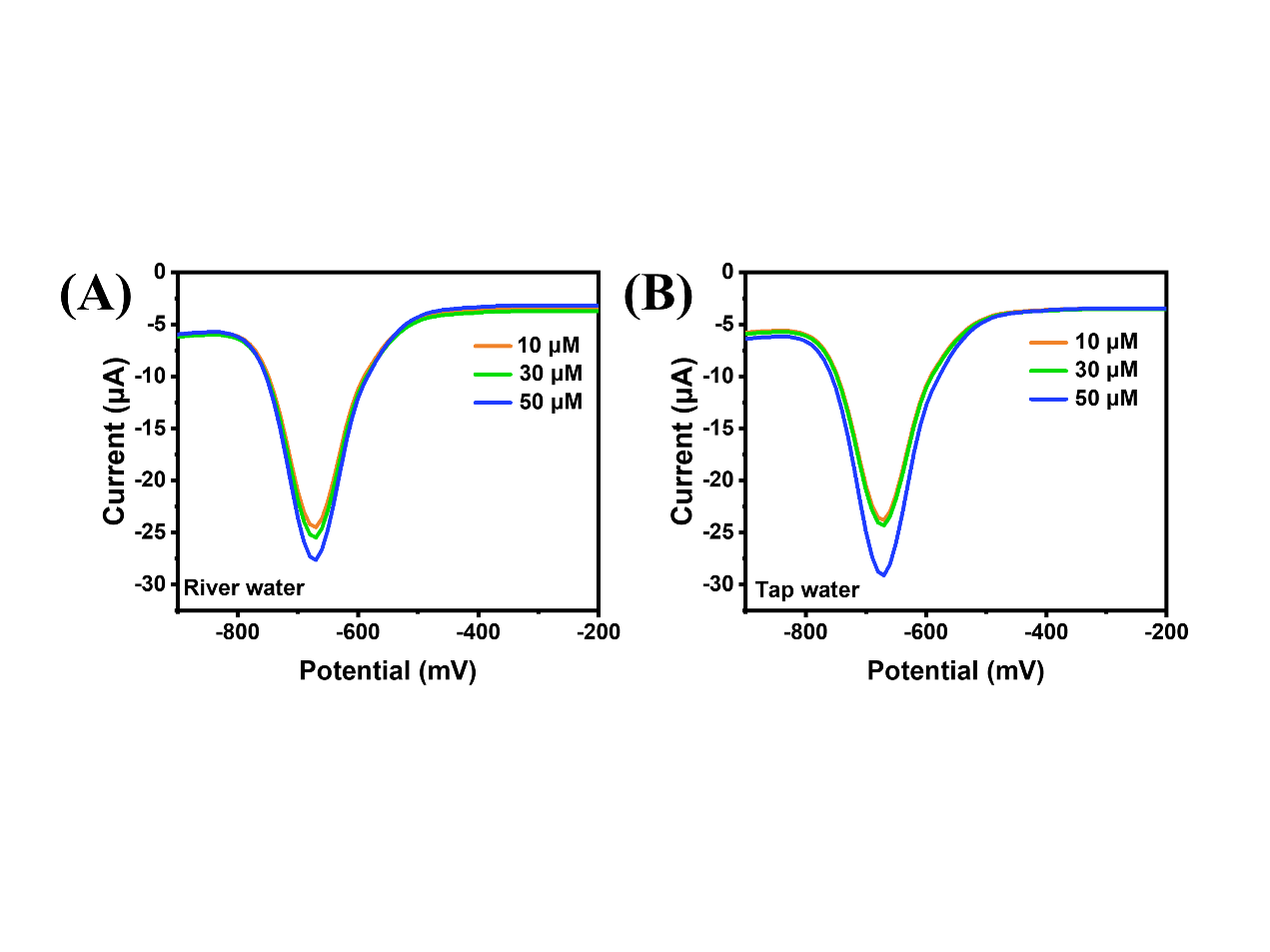


**FigS3**. (A). DPV of MnMoO_4_/g-C_3_N_4_/CHIT/GCE for different concentrations of TNZ in river water; (B). DPV of MnMoO_4_/g-C_3_N_4_/CHIT/GCE for different concentrations of TNZ in tap water.

**Scheme**

**+4H^+^, +4e^-^**

**Scheme**.**S1** Possible electro-reduction mechanism of TNZ on MnMoO_4_/g-C_3_N_4_/CHIT/GCE electrode


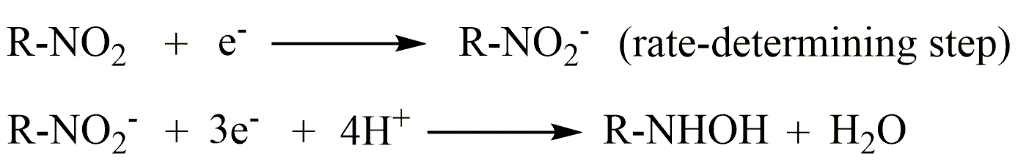


**Scheme.S2.** Proposed electrochemical reduction mechanism of TNZ on MnMoO_4_/g-C_3_N_4_/CHIT/GCE.

**Table**

Table S1 Pore properties of g-C_3_N_4_, MnMoO_4_/g-C_3_N_4_ and MnMoO_4_/g-C_3_N_4_/CHIT

| Sample | Specific surface area/(m^2^·g^-1^) | Pore volume（cm^3^/g） | Average aperture（nm） |
| --- | --- | --- | --- |
| g-C_3_N_4_ | 9.27 | 0.05 | 22.58 |
| MnMoO_4_/g-C_3_N_4_ | 30.47 | 0.19 | 24.91 |
| MnMoO_4_/g-C_3_N_4_/CHIT | 26.98 | 0.17 | 24.53 |

| Samples | Added (μM) | Found (μM) | Recovery (%) | RSD (%) (n=3) |
| --- | --- | --- | --- | --- |
| Songhua River | 10 | 10.96 | 109.6 | 1.0 |
|  | 30 | 28.98 | 96.6 | 2.0 |
|  | 50 | 47.33 | 94.7 | 2.0 |
| Tap water | 10 | 9.65 | 96.5 | 1.7 |
|  | 30 | 31.98 | 106.6 | 1.0 |
|  | 50 | 50.31 | 100.6 | 2.6 |

Table S2 HPLC detection of TNZ in river water and tap water
